# Supplementary material for: Surface Microbiome Profiling of Dental Elevators Using Third‐Generation Sequencing: Implications for Infection Control in Dental Practice
Source: Microbiologyopen. 2025 Dec 8;14(6):e70178. doi: 10.1002/mbo3.70178 (PMC12685768; doi:10.1002/mbo3.70178)
Supplement: Supplementary file 1 — customer_backup. [file MBO3-14-e70178-s001.zip › customer_backup/customer_backup/taxa_summary/krona/groups/treat/M15.Krona.html]

Javascript must be enabled to view this page.

members
magnitude
magnitudeUnassigned

M15.krona

55140

55013

571

526

522

214

214

214

55

55

55

253

253

253

4

4

4

4

45

45

45

45

0

0

1

44

0

0

0

0

0

159

159

159

159

6

6

8

8

24

24

12

12

109

109

229

219

0

0

0

0

7

7

4

4

1

1

0

0

0

0

0

2

2

212

11

11

11

3

1

1

2

2

2

0

0

2

2

180

180

180

10

4

4

1

1

0

0

5

0

5

6

6

6

0

0

10

0

0

0

0

0

0

0

10

1

1

1

1

1

1

2

2

2

2

2

2

4

4

4

4402

26

26

0

0

0

26

15

15

0

9

1

8

0

0

2

2

1558

72

72

72

72

1417

0

0

0

89

89

89

30

3

3

11

11

16

16

1298

53

46

7

40

40

201

181

20

605

77

120

140

268

151

151

248

87

161

69

69

69

57

12

465

465

465

417

417

48

48

1825

1825

563

4

4

53

53

1

1

3

3

82

82

17

17

403

393

10

40

26

26

14

14

170

170

170

1052

1052

17

881

34

120

0

0

527

521

486

59

59

427

427

4

4

4

19

19

19

12

12

12

3

3

3

3

0

0

0

0

3

3

3

3

1

1

1

1

1

11

11

11

11

11

11

0

0

0

0

0

0

0

0

0

0

0

0

0

0

33

33

33

6

6

6

6

6

6

11

11

11

10

10

10

0

0

0

474

5

5

5

1

1

2

2

1

0

1

1

0

1

26

26

26

26

26

443

443

443

1

1

0

439

47

0

389

0

3

2

2

1

0

1

540

85

85

11

11

11

74

74

74

136

127

83

83

83

5

5

5

39

39

39

9

9

9

4

5

317

150

149

3

1

2

0

146

1

0

145

0

1

1

1

0

1

1

1

1

0

0

0

0

0

161

0

0

0

0

0

0

0

0

101

5

5

85

85

11

11

44

20

1

19

0

0

0

24

24

16

16

4

12

5

5

0

0

5

5

0

0

0

0

0

0

0

2

2

2

2

2

17878

1278

1

1

1

1

15

0

0

0

15

0

0

0

0

0

0

0

0

15

1

14

0

0

0

0

10

10

9

3

6

0

0

1

1

11

0

0

0

11

0

0

0

0

0

0

11

11

0

0

0

1

1

0

0

0

0

1

0

0

1

0

13

13

13

13

0

0

0

69

69

0

0

9

5

0

0

4

0

3

3

0

0

0

0

0

0

0

0

0

0

3

3

2

0

0

0

1

0

0

1

1

1

22

0

13

4

5

0

0

0

25

0

0

0

1

0

1

0

0

1

0

1

0

5

0

0

0

0

2

9

0

1

0

0

3

0

0

0

0

0

1

4

0

2

0

2

16

0

0

0

16

0

0

16

16

517

156

0

0

94

94

0

0

0

62

40

22

0

0

0

0

0

0

0

0

0

0

4

0

0

0

4

4

0

0

0

0

0

0

0

0

0

0

0

0

0

0

0

2

2

2

0

0

0

1

1

1

107

10

10

0

0

1

0

1

9

8

1

0

0

0

4

4

30

21

9

42

42

0

0

8

0

3

5

0

0

0

0

0

0

3

0

1

0

0

0

0

0

0

0

0

0

2

0

0

3

0

0

1

1

1

1

0

0

1

1

0

0

0

2

2

2

0

0

0

0

0

0

83

0

0

0

0

71

57

14

9

9

3

0

3

0

0

90

78

1

77

12

0

12

10

0

0

0

1

1

3

3

0

0

0

0

0

0

0

0

0

0

0

6

0

6

1

1

0

1

58

26

26

0

0

0

0

0

0

0

0

0

0

29

0

2

0

0

18

0

0

0

0

0

9

0

0

0

0

0

1

0

0

0

1

0

0

0

0

0

0

2

2

0

0

0

0

0

0

85

1

0

0

1

1

84

0

0

0

0

0

1

0

1

82

81

1

0

0

1

1

0

0

0

0

1

1

1

1

168

168

11

4

6

1

154

41

30

83

3

3

1

1

1

1

0

0

0

0

0

0

0

0

1

1

1

0

1

0

0

0

0

17

6

6

6

11

11

11

0

0

0

0

0

0

0

24

24

0

0

1

0

1

6

6

12

9

1

0

1

0

1

0

0

0

0

3

1

2

0

0

0

2

0

1

1

0

0

0

0

0

0

0

0

87

0

0

0

75

75

75

0

0

0

0

0

0

1

1

1

0

0

0

0

0

0

0

0

0

11

11

11

241

241

12

12

0

0

4

4

8

8

19

8

11

0

0

19

3

6

10

14

14

3

3

5

5

1

1

2

2

0

12

12

0

0

3

0

0

2

0

1

33

3

8

22

0

0

0

0

106

0

106

0

16600

11

11

11

8

3

6

6

0

0

6

6

0

2

2

2

2

0

0

0

9063

8

8

8

0

0

0

0

0

0

0

0

0

113

0

0

0

0

0

0

0

0

0

113

113

0

0

0

4

4

4

5

5

5

0

0

4954

1

1

0

0

4845

4845

94

8

24

44

18

14

0

0

0

0

0

6

0

2

0

3

0

0

0

0

0

3

23

0

0

2

2

1

0

0

1

0

0

13

13

7

7

0

0

0

0

7

0

0

0

0

0

0

6

0

1

5

1

1

0

0

0

0

1

1

1

0

0

0

0

0

0

0

0

0

1

1

1

3937

0

0

0

0

0

0

0

0

2

2

0

0

1

0

1

0

11

7

4

0

0

1386

1

1385

0

0

1

0

1

275

0

275

0

1

1

4

0

4

2256

2256

0

0

10

2

2

8

0

8

2540

2

1

0

1

1

1

0

0

2538

2494

836

1658

7

7

37

37

3

3

3

3

1082

635

635

635

447

447

447

0

0

0

0

0

0

0

0

0

156

143

143

86

41

16

13

13

13

11

11

8

8

3

3

457

0

0

0

7

0

0

0

0

0

3

3

0

0

4

0

1

0

3

2

2

2

13

7

7

6

6

20

20

20

0

0

0

0

9

9

9

0

0

0

9

9

0

0

7

2

396

38

27

1

10

57

30

27

271

136

81

54

0

0

30

30

1

1

1

0

0

0

0

17

17

17

17

28

17

4

4

13

13

0

0

11

11

11

48

48

48

48

213

109

109

109

104

5

5

36

36

63

10

53

152

152

152

152

1570

0

0

0

0

0

1

1

1

995

988

876

112

7

7

2

2

2

224

196

196

22

22

6

6

0

0

2

2

0

2

0

0

0

0

0

0

2

2

2

344

205

205

0

0

138

138

0

0

0

0

0

0

0

1

1

0

3

3

3

3

61

30

30

30

31

31

31

0

0

0

0

0

0

0

0

0

0

0

24

24

24

24

20

11

11

11

9

9

9

80

1

0

0

0

0

0

1

1

0

0

0

0

0

79

74

74

0

0

0

0

1

1

0

0

0

0

0

0

4

0

0

0

0

0

0

1

0

0

0

1

2

0

10

4

4

4

6

6

6

26

26

26

26

49

39

39

39

10

10

10

11

11

11

11

648

648

0

0

0

0

2

2

0

0

0

0

0

0

646

144

58

444

0

0

6

6

6

6

0

0

0

0

198

15

15

15

32

32

32

151

151

151

91

91

59

7

52

32

32

14

14

14

8

6

640

405

405

0

0

0

405

2

2

0

0

0

0

0

0

400

1

399

0

0

0

0

3

3

0

0

235

7

5

5

5

2

2

2

0

0

0

0

0

0

0

0

0

0

186

0

0

0

0

0

0

0

0

0

0

0

0

0

0

8

8

0

8

17

17

17

161

1

0

1

160

158

0

2

0

0

0

0

0

0

3

3

3

3

11

11

11

0

0

1

10

18

12

12

12

6

6

6

1

1

1

1

9

9

9

9

0

0

0

0

0

0

6

6

6

6

6

0

3

0

3

0

0

0

0

0

0

5456

7

7

7

7

7

5269

108

0

0

0

32

0

0

0

7

1

3

3

0

0

0

0

0

1

0

1

0

0

0

0

0

9

0

1

1

1

2

3

0

1

0

0

0

0

0

0

0

0

0

0

0

1

0

0

1

2

2

0

0

0

0

0

0

12

12

76

73

63

0

0

10

3

3

216

0

0

0

214

8

8

0

0

0

0

5

5

1

1

0

0

0

0

4

4

21

21

0

0

0

0

0

0

0

0

0

0

0

12

12

7

7

43

43

5

5

70

70

31

31

7

7

0

0

0

0

0

0

0

0

0

0

0

2

0

0

0

0

2

2

0

0

4742

43

43

43

0

0

0

0

0

0

0

0

1663

2

2

3

3

413

413

1245

1245

868

21

0

21

0

0

175

0

0

114

0

61

0

0

672

0

8

7

2

0

320

60

275

0

0

0

0

0

21

1

1

0

0

20

0

1

19

129

48

48

81

81

0

0

0

339

339

0

12

18

22

84

0

0

0

1

0

180

0

10

1

1

2

8

164

13

13

0

0

0

151

55

13

7

66

1

9

951

0

0

0

111

0

111

82

29

1

52

0

0

111

111

0

0

0

0

130

0

0

41

0

34

0

0

0

0

0

0

55

0

0

0

0

0

517

223

23

0

0

0

1

0

0

208

0

52

0

0

0

10

0

76

76

76

62

62

62

415

415

10

55

0

350

0

0

11

11

11

0

0

0

28

0

0

0

0

0

0

0

0

0

0

0

0

26

12

12

6

6

8

8

0

0

0

2

2

2

172

4

0

0

1

1

0

0

3

0

3

0

56

0

0

56

39

13

4

0

0

0

0

4

1

1

0

0

1

0

1

1

1

1

1

0

0

0

0

62

0

0

0

0

62

0

62

0

46

4

4

6

2

0

1

2

1

0

0

0

0

0

0

6

4

2

30

0

0

0

0

1

0

10

0

1

12

4

1

1

0

3

3

3

3

65

65

65

0

0

65

65

1

1

1

1

1

0

0

0

114

114

33

33

7

26

81

14

14

67

67

0

0

0

0

259

257

11

11

7

7

4

2

2

0

0

0

128

28

28

28

100

12

12

88

88

3

3

3

3

5

5

5

5

6

6

6

6

0

0

0

104

104

104

104

2

2

2

0

0

0

1

1

1

0

1

0

0

0

0

195

195

195

195

182

182

13

13

2913

1252

1252

0

0

0

1128

663

99

0

562

2

465

256

0

209

0

124

0

0

2

0

1

0

1

1

1

2

2

3

3

0

0

0

74

74

0

30

0

29

1

12

0

12

0

0

0

86

86

86

86

86

965

783

781

57

57

150

150

36

36

0

0

2

2

471

471

65

65

2

2

0

2

3

0

0

0

1

1

1

1

1

1

1

1

1

0

0

0

0

0

0

0

0

0

0

0

0

0

0

0

0

0

0

0

0

0

0

0

0

0

0

0

0

0

179

73

4

1

3

19

14

5

50

2

41

7

47

47

3

1

43

35

1

0

1

5

0

5

29

16

13

24

8

8

0

0

16

16

80

56

1

1

1

20

13

0

3

0

2

3

5

0

0

0

0

0

0

4

0

0

4

1

0

0

1

2

2

35

1

1

7

7

27

27

0

0

0

0

24

12

12

2

8

2

12

0

0

1

1

11

11

526

0

0

0

0

0

0

0

0

0

0

0

0

0

0

0

0

3

3

3

3

2

2

0

0

0

0

0

0

0

0

0

0

0

0

0

0

0

0

0

0

1

0

1

0

1

1

0

0

0

0

0

0

1

1

0

0

0

0

1

1

0

0

6

2

2

0

2

0

0

0

0

2

2

1

0

0

1

2

2

0

2

0

0

0

0

0

0

188

188

0

0

0

188

80

14

90

0

1

0

0

3

0

0

15

5

0

0

2

2

3

3

0

0

0

0

0

0

0

0

0

0

0

0

0

0

0

0

10

3

3

2

2

5

5

1

0

0

0

0

0

0

1

0

0

1

1

0

81

81

2

2

0

0

79

1

12

0

0

0

42

3

0

0

0

1

20

0

0

0

0

0

0

0

0

0

0

0

0

0

0

0

0

0

0

0

0

49

10

7

7

0

0

0

0

0

0

0

0

0

0

0

0

0

0

1

0

1

0

2

2

39

7

0

1

6

0

0

31

18

0

2

1

0

0

0

3

1

0

0

0

0

0

3

0

0

0

0

3

0

0

0

0

0

1

0

1

0

0

3

3

0

0

0

0

2

2

0

0

0

0

0

1

0

0

0

0

1

0

0

0

0

0

0

0

0

0

0

0

0

177

63

0

0

3

3

24

0

24

0

0

2

1

1

21

0

4

3

2

12

0

0

0

0

13

13

9

0

0

1

1

0

0

8

0

8

0

0

0

0

0

0

0

0

0

0

14

12

12

0

0

2

2

91

4

4

0

0

1

1

3

1

2

79

79

0

0

0

0

0

0

0

0

0

0

0

0

3

3

1

0

0

1

0

0

0

0

0

0

0

0

0

0

0

0

0

0

0

0

4

4

4

4

1

2

1

0

78

70

70

70

4

4

0

0

0

0

0

0

0

0

0

0

0

0

0

0

0

0

0

0

0

0

0

25

4

21

27

27

14

14

8

8

8

8

8

0

0

0

0

0

992

992

992

992

992

992

31

31

31

27

0

0

8

8

16

16

3

1

2

4

4

2

0

0

0

2

0

0

0

0

278

278

278

165

165

1

12

152

0

0

0

0

0

0

0

4

4

4

0

0

0

109

109

109

3

0

0

0

0

0

0

0

0

0

0

0

0

0

0

0

3

3

3

3

3

0

0

0

0

0

0

1

1

1

1

1

1

0

0

0

1198

59

56

56

56

56

3

0

0

0

0

0

0

0

0

0

0

3

0

0

1

1

0

0

2

2

2

1

1

1

0

1

0

0

0

0

0

0

0

0

0

1

1

0

0

1

0

0

1

0

0

0

0

0

0

0

0

0

0

0

0

0

0

0

0

0

0

0

0

0

0

0

0

1064

1064

1064

391

391

673

1

255

0

3

0

0

0

414

61

61

61

61

61

12

5

1

1

1

3

3

3

1

1

1

7

7

7

7

146

0

0

0

0

0

0

0

0

0

0

0

0

0

4

0

0

0

0

0

0

0

0

0

0

0

0

0

0

0

4

4

4

4

0

0

0

0

0

0

0

0

0

0

0

9

0

0

0

0

9

9

9

9

0

0

0

0

0

0

0

0

0

0

8

8

8

8

8

125

125

0

0

0

0

0

0

0

0

0

0

0

0

0

0

0

0

0

125

0

0

1

0

1

124

124

0

0

0

0

0

0

0

0

0

0

0

0

21

21

21

8

8

8

13

13

13

3

3

3

3

3

3

0

0

0

0

0

0

0

0

0

0

0

0

0

0

37

27

1

1

0

0

1

1

0

0

0

0

0

26

25

25

25

0

0

0

0

0

0

0

0

0

0

0

0

1

1

1

7

7

7

7

7

3

3

3

0

0

3

0

3

0

0

0

0

0

24

24

24

21

21

0

0

21

3

0

0

0

3

0

0

0

0

0

1

2

51

51

51

51

51

51

0

0

18160

5888

3

3

0

0

0

2

2

1

1

0

0

0

0

0

19

19

17

17

2

2

16

0

0

0

0

0

0

16

16

16

0

0

11

11

11

0

11

0

2552

2551

3

3

0

0

0

0

0

0

0

60

23

37

21

21

0

303

33

269

1

0

0

3

0

3

10

10

0

0

0

0

1

1

0

0

107

107

0

0

119

0

101

18

138

138

49

0

2

3

0

2

1

0

0

0

19

3

0

5

0

2

0

5

3

1

3

0

0

0

0

17

0

17

0

0

25

8

16

1

303

95

4

0

198

0

6

368

0

0

0

2

359

0

0

7

0

3

3

369

5

0

196

168

0

65

65

55

55

0

0

0

0

0

0

0

0

0

0

0

0

8

8

6

6

71

0

7

47

17

20

20

1

1

50

50

2

2

4

4

7

3

4

0

0

0

53

4

49

1

1

0

309

2

154

1

10

1

55

64

3

0

5

8

6

0

0

0

1

1

1

0

0

0

0

2090

903

0

0

8

4

4

0

0

0

0

0

0

166

90

76

0

0

726

720

0

0

0

6

3

3

0

0

0

1186

3

3

0

0

1112

1112

0

0

0

0

2

2

69

69

0

1

0

0

0

0

0

0

0

1

1

0

0

0

0

158

158

0

0

0

0

157

78

0

0

0

72

0

7

1

1

232

3

3

3

0

0

0

0

0

0

216

216

216

13

13

13

0

0

0

0

0

0

0

0

0

800

693

13

6

7

0

0

24

24

1

1

0

45

45

0

0

10

0

10

0

0

1

1

0

0

2

2

0

0

0

380

4

360

0

16

0

11

0

1

0

9

0

0

1

0

0

88

0

88

50

50

13

0

13

55

0

0

0

55

13

13

13

0

0

0

30

2

0

2

26

25

0

0

1

2

2

0

0

0

1

1

1

1

0

0

1

1

62

13

3

0

0

0

10

0

5

5

0

0

5

1

4

0

0

0

0

11

5

3

1

2

26

26

0

0

0

2

1

1

0

0

0

7

5

5

5

0

0

0

2

2

2

0

0

0

0

0

0

12002

0

0

0

0

968

234

0

0

0

0

0

0

136

3

1

0

0

132

1

0

1

95

95

2

2

0

0

0

0

734

0

0

0

0

0

0

0

734

2

0

3

1

2

4

0

0

0

15

3

34

3

0

1

0

666

0

0

0

0

0

0

0

0

0

0

0

0

0

3275

210

8

8

0

0

49

49

31

16

0

0

0

11

4

112

112

2

2

8

3

5

0

0

3065

14

14

285

285

20

5

15

5

0

5

2240

2232

8

3

3

31

31

35

35

17

3

14

27

27

381

381

0

0

4

4

3

3

0

0

0

0

0

0

0

0

68

49

1

1

0

0

48

4

44

19

19

1

1

17

89

89

89

89

0

53

53

0

0

0

0

5

0

0

0

0

1

0

0

0

1

3

0

0

0

0

0

0

0

0

0

0

0

0

0

0

0

0

46

46

2

2

0

0

0

0

47

2

2

2

0

0

0

0

0

0

45

45

45

8

8

8

6

2

7494

2

2

2

0

3

2

2

0

0

0

0

1

1

0

0

1702

1701

15

709

1

0

3

0

7

955

3

2

0

0

6

1

1

0

10

10

1

9

5757

0

0

0

0

299

293

1

5

60

60

0

0

2150

0

1074

126

0

0

950

14

14

2192

1899

0

0

0

0

293

9

9

1022

0

0

1022

3

2

1

7

7

1

1

0

0

0

0

20

0

0

18

18

2

2

0

0

0

0

0

0

0

0

0

0

0

0

0

0

0

0

0

0

0

0

0

0

0

0

0

0

0

0

0

0

0

0

269

108

108

108

108

0

0

161

1

1

1

13

11

11

0

0

1

1

0

0

0

1

0

1

0

0

0

0

0

0

0

142

24

24

0

0

0

118

0

111

0

7

0

0

0

5

5

5

1

1

1

1

1

107

24

19

0

0

0

19

0

0

19

19

0

0

0

0

5

0

0

0

5

5

5

0

0

0

1

0

0

0

0

0

0

0

0

0

0

0

1

1

1

1

77

17

17

17

17

14

14

14

2

12

0

0

0

0

0

4

0

0

0

4

4

4

42

42

27

27

0

0

15

15

0

0

0

0

0

0

0

0

0

0

5

5

5

5

5

11

11

11

11

11

8

3

6

6

6

6

6

5

1

96

96

96

96

96

96

1

1

1

0

0

0

0

0

0

1

1

1

0

0

0

3

3

3

3

3

3

0

0

0

0

0

0

0

127

0

0

0

0

0

0

0

0

0

0

0

0

0

0

0

0

0

127

127

127

127

127

127

0

0

0

0

0

0
